# Useful condition of chromoendoscopy with indigo carmine and acetic acid for identifying a demarcation line prior to endoscopic submucosal dissection for early gastric cancer

**DOI:** 10.1186/s12876-016-0483-7

**Published:** 2016-07-19

**Authors:** Norifumi Numata, Shiro Oka, Shinji Tanaka, Yoshikazu Yoshifuku, Tomohiro Miwata, Yoji Sanomura, Koji Arihiro, Fumio Shimamoto, Kazuaki Chayama

**Affiliations:** Department of Gastroenterology and Metabolism, Graduate School of Biomedical Sciences, Hiroshima University, Hiroshima, Japan; Department of Endoscopy, Hiroshima University Hospital, Hiroshima, Japan; Department of Pathology, Hiroshima University Hospital, Hiroshima, Japan; Faculty of Human Culture and Science, Prefectural University of Hiroshima, Hiroshima, Japan

**Keywords:** Gastric cancer, Endoscopic submucosal dissection, Chromoendoscopy, Acetic acid

## Abstract

**Background:**

Identifying a precise demarcation line (DL) is indispensable for pathological complete en bloc endoscopic submucosal dissection (ESD) for early gastric cancer (EGC). We evaluated the useful condition of chromoendoscopy with indigo carmine and acetic acid for marking dots around lesions before ESD for EGC.

**Methods:**

We examined 98 consecutive patients with 109 intramucosal EGCs (mean diameter, 17.8 ± 12.4 mm; main histologic type, 96 intestinal and 13 diffuse) resected by en bloc ESD after chromoendoscopy with indigo carmine and acetic acid between December 2012 and February 2014. The DL was identified by this technique just before ESD (mean chromoendoscopy observation time, 71.6 s); subsequently, marking dots were placed around the EGC. EGCs were classified into two groups: useful for identifying the DL or useless. Clinicopathological characteristics and clinical outcomes were evaluated in each group.

**Results:**

Forty-two of the 109 cases (38.5 %) were determined useful for chromoendoscopy with indigo carmine and acetic acid. Multivariate analysis with logistic regression showed that macroscopic type (protruded or flat elevated-type) and atrophic border (the oral side of tumor) were independently associated with the usefulness of chromoendoscopy using indigo carmine and acetic acid for identifying the DL of EGCs (*P* < 0.05). The histologically positive horizontal margin after ESD was 0 % (0/42) in useful cases, and 7.5 % (5/67) in useless cases.

**Conclusions:**

Before ESD, chromoendoscopy with indigo carmine and acetic acid can be used for creating precise markings in protruded or flat elevated-type EGC or at the atrophic border on the oral side of EGCs.

## Background

Endoscopic submucosal dissection (ESD) is a widely accepted treatment for early gastric cancer (EGC), and it can be performed regardless of tumor size, location, or fibrosis [1–11].

There has been an increase in the number of ESDs for EGC and a corresponding increase in the number of en bloc specimens with a positive horizontal margin (HM). In order to reduce the incidence of positive HMs, it will be important to improve the diagnostic performance of magnifying endoscopy with narrow band imaging (ME-NBI) for identifying a demarcation line (DL), as well as for improving ESD procedure itself and specimen handling [12]. Identification of a precise DL is indispensable for performing pathological complete en bloc ESD for EGC. In Japan, ME-NBI is a standardized technique for determining DL of EGCs during ESD [13]. However, ME-NBI needs to be close to the lesion and takes a long time to perform when the lesion is of a large size or if the lesion is in a position that is difficult to access with the vertical approach of the scope.

Chromoendoscopy, which has been used in combination with indigo carmine and acetic acid, has been reported as a novel technique for identifying a DL. This technique improves the diagnostic yield for delineating the margin of EGGs [14–16] or diagnosis of gastric neoplasia [17], and enables observation without magnification. However, the useful condition of chromoendoscopy with indigo carmine and acetic acid for marking dots around lesions before ESD for all types of ECGs remains unclear.

Therefore, the aim of the present study was to determine patient- and tumor-related factors for identifying the DL by chromoendoscopy with indigo carmine and acetic acid for EGCs.

## Methods

### Patients

The study group comprised 98 consecutive patients with 109 intramucosal EGCs resected by en bloc ESD after chromoendoscopy with indigo carmine and acetic acid at Hiroshima University Hospital between December 2012 and February 2014. We classified the lesions as either useful or useless, according to the visibility of the DL using chromoendoscopy with indigo carmine and acetic acid. The indications for ESD were based on the expectation that the procedure would be curative, as indicated in the Japanese Classification of Gastric Carcinoma issued by the JGCA [18]. All patients provided written informed consent for the ESD procedure and follow-up assessment. The study was conducted in accordance with the Declaration of Helsinki and was approved by the Institutional Review Board of the Hiroshima University Hospital.

### Evaluation of chromoendoscopy with indigo carmine and acetic acid images

**Fig. 1:**
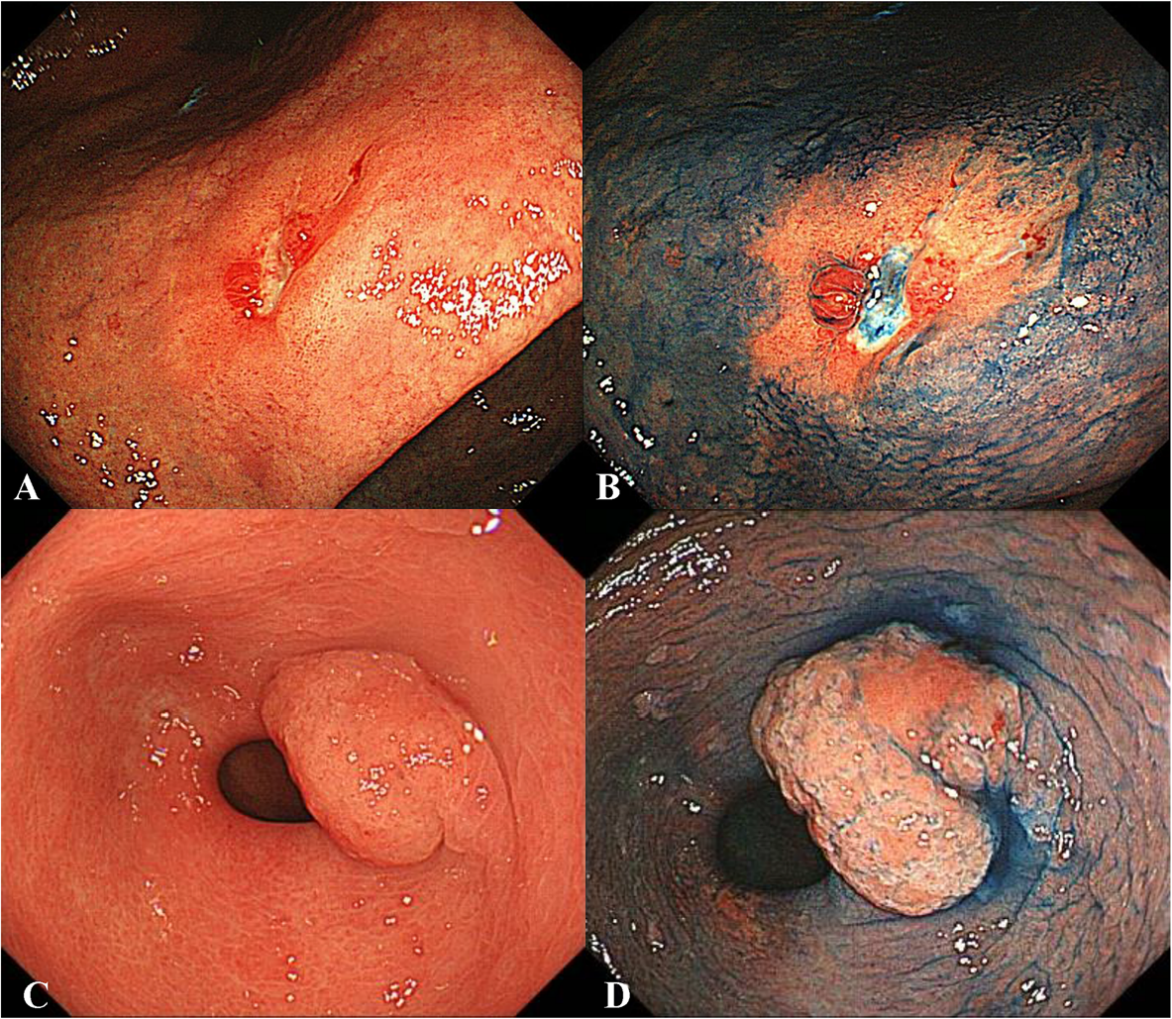
Example of useful case and useless case. **a** Reddish flat lesion with small ulcer is shown at the lesser curvature of the body by conventional endoscopy; **b** After dying indigo carmine and acetic acid, the DL became more clearly (this image scored 8, and classified to useful case); **c** A normochromic protruded lesion is shown at the posterior wall of the antrum by conventional endoscopy, and the DL can be recognized as the rising portion of the protruded lesion; **d** After dying indigo carmine and acetic acid, the DL seems to be not obviously improved (this image scored 4, and classified to useless case)

Four expert endoscopists, who experienced in more than 200 gastric ESD cases, participated in our study evaluation. The conventional endoscopic images were presented to each of the physicians in random order and blinded for comparison with the chromoendoscopy with indigo carmine and acetic acid images. Physicians scored the chromoendoscopy with indigo carmine and acetic acid images for visibility of the DL according to the following scale: +2 (improved visibility), +1 (some- what improved visibility), 0 (visibility equivalent to that of conventional visibility), −1 (somewhat decreased visibility), and −2 (decreased visibility). The 4 physicians’ scores for each chromoendoscopy with indigo carmine and acetic acid image were tallied. If an image had a total score of 7 or 8, the image was considered useful for determining the while a score between 6 or less indicated that it was not useful (Fig. 1).

### ESD procedure

ESD was performed as previously reported [5, 6, 8, 9, 19]. We identified the DL by white light endoscopy and chromoendoscopy with indigo carmine and acetic acid just before ESD (mean chromoendoscopy observation time, 71.6 s). For chromoendoscopy with indigo carmine and acetic acid, we initially dyed the tumor with 1.5 % acetic acid, followed by indigo carmine 30 s later. If the chromoendoscopy with indigo carmine and acetic acid were not useful to determine the DL, we used ME-NBI. After identifying the DL, marking dots were placed around the lesion using an argon plasma coagulation probe. An extra marking dot was placed on the DL of the tumor and confirmed by the pathological analysis.

In all patients, the presence or absence of endoscopic submucosal fibrosis findings were classified according to a previously reported classification [8, 20]. Based on observations at the time of injection of sodium hyaluronate with indigo carmine, lesions were classified as F0 (no fibrosis; manifests as a blue transparent layer), F1 (mild fibrosis; appears as a white web-like structure in the blue submucosal layers), and F2 (severe fibrosis, appears as a white muscular structure without a blue transparent layer in the submucosal layers). Poor control of bleeding during ESD indicated the frequent need for coagulation with hemostatic forceps [19].

Follow-up endoscopy after ESD was performed immediately after positive HM was identified by pathologic diagnosis. If there was no local recurrence, surveillance endoscopy was performed at 3–6, 12, and every 12 months thereafter, respectively. If the HM was negative, follow-up endoscopy was performed every 12 months. Biopsy of the ESD scar site analysis was always performed, whether or not local recurrent tumor was observed endoscopically.

### Pathologic evaluation of resected specimens

Resected specimens were fixed in formalin solution and cut into 2-mm thick sections. Histopathological diagnosis was based on the Japanese Classification of Gastric Carcinoma [21]. According to the Japanese Gastric Cancer Association (JGCA) [18], the absolute indication for curative ESD are well- or moderately differentiated intramucosal adenocarcinoma tumors that are ≤ 2 cm in diameter without ulceration, and lymph node metastasis and lymphatic vessel invasion. The expanded indication added well- or moderately differentiated intramucosal adenocarcinoma tumors that are > 2 cm in diameter without ulceration, well- or moderately differentiated adenocarcinoma lesions that are ≤ 3 cm in diameter with submucosal invasion of < 500 μm without ulceration, and well- or moderately differentiated adenocarcinoma with ulceration.

### Immunohistochemistry for mucin phenotype of EGC

The immunohistochemical analysis was performed according to the method described by Sasaki et al. [22], with minor modifications. Immunohistochemistry for anti-human gastric mucin (HGM), MUC2, MUC6, and CD10 was performed on formalin-fixed, paraffin-embedded tissues cut into serial 4 μm sections, respectively. Sections were deparaffinized and rehydrated in phosphatase-buffered saline (PBS, PH 7.2) and microwaved twice for 5 min each in Dako REAL Target Retrieval Solution (Dako) for antigen retrieval. The slides were placed in a humidified chamber and incubated with protein blocking solution (5 % normal horse serum and 1 % normal goat serum in PBS) for 20 min at room temperature. Primary antibodies were applied to the slides and incubated overnight in humidified boxes at 4 °C. The primary antibodies that were used included the HGM monoclonal antibody (Novocastra, Newcastle, UK) and anti-Muc6 (Novocastra) for gastric mucin as well as the anti-Muc2 (Novocastra) and CD10 antibody (Novocastra) for intestinal mucin. After incubation for 1 h at room temperature with corresponding peroxidase-conjugated secondary antibodies, a positive reaction was detected by exposure to stable 3,3′-diaminobenzidine for 5 to 10 min. Slides were counterstained with hematoxylin for visualization of the nucleus. The results of immunostaining were considered positive if more than 10 % of tumor cells were stained for each marker. According to the results, tumors were classified into the gastric (G-type), intestinal (I-type), and mixed phenotypes (M-type).

### Outcome measurements

Clinicopathological characteristics (sex, age, tumor location [U or M/L], tumor diameter, macroscopic type of tumor [elevated, 0-I & 0-IIa & 0-IIb; depressed, 0-IIa + IIc & 0-IIc], color of tumor [reddish/normal or pale], presence of atrophic border [oral side /anal side of tumor], presence of intestinal metaplasia around tumor [present/absent], histologic type of tumor [intestinal/diffuse], mucin phenotype of tumor [G type/I type /M type], depth of tumor [intramucosal/submucosal], presence of ulceration [positive or negative], visibility of the DL with indigo carmine only) and clinical outcomes (complete en-bloc resection, degree of fibrosis during ESD, histologically positive HM, local recurrence rate, procedure time, poor control of bleeding during ESD, perforation, post-ESD bleeding) were compared between the two groups.

### Statistical analysis

Differences between the two groups were analyzed using the χ2-test with Yates’ correction, Fisher’s exact test, or Student’s t-test, respectively. A *P* value of < 0.05 was considered significant.

## Results

**Fig. 2:**
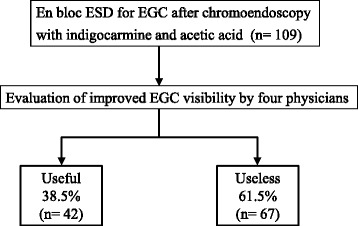
Flowchart showing the 109 ESD cases included in this study. ESD, endoscopic submucosal dissection; EGC, early gastric cancer

Among the cases of chromoendoscopy with indigo carmine and acetic acid for marking dots around lesions before ESD for EGCs, 38.5 % (42/109) were classified as useful (Fig. 2). The clinicopathological characteristics of the EGCs are shown in Table 1. Protruded or flat elevated type tumors (including 4 0–IIb lesions), or an atrophic border, which were located in the oral side of tumor, and the intestinal type of histology, were significantly more frequently identified in cases wherein chromoendoscopy images with indigo carmine and acetic acid were useful for identifying the DL. No type 0–IIb lesions were observed among the useless cases. There were no significant differences in the mucin phenotype of the differentiated type gastric cancers. Clinical outcomes of ESD for EGCs are demonstrated in Table 2. The histologically positive horizontal margin after ESD was 0 % (0/42) in useful cases, and 7.5 % (5/67) in useless cases, respectively, but was not statistically significant. The tumor margin of these 5 positive HM cases could not be evaluated because of burn damage caused by ESD. Furthermore, local recurrence did not occur. Multivariate analysis with logistic regression revealed that both “protruded or flat elevated type” and “atrophic border which exists in the oral side of EGC” were significant tumor-related factors for identifying the DL by chromoendoscopy using indigo carmine and acetic acid (Table 3).

**Table 1:** Characteristics of EGCs between useful and useless groups

Clinicopathological features	Useful	Useless	*P* value
Number of cases	42	67	
Sex (Male/Female)	34/8	53/14	N.S.
Age (years)	71.7 ± 7.4	68.6 ± 10.8	N.S.
Tumor location (U/M/L)	9/16/17	12/18/37	N.S.
Tumor diameter (mm, mean ± SD)	17.5 ± 13.8	19.7 ± 10.1	N.S.
Macroscopic type (protruded or flat elevated/depressed)	23/19	18/49	<0.01
Color (reddish/normal & pale)	12/30	31/36	N.S.
Atrophic border (oral side/anal side of tumor)	38/4	46/21	<0.01
Intestinal metaplasia around tumor (present/absent)	10/32	12/55	N.S.
Histologic type (intestinal/diffuse)	41/1	55/12	<0.05
Mucin phenotype (G-type/I-type/M-type)	14/17/11	18/32/17	N.S.
Depth (intramucosal/submucosal)	38/4	53/14	N.S.
Ulceration (+/−)	1/41	8/59	N.S.
Visibility of DL with indigo carmine only (good/bad)	4/38	13/54	N.S.

*U* upper stomach, *M* middle stomach, *L* lower stomach, *G-type* Gastric type, *I-type* Intestinal type, *M-type* Mix type

**Table 2:** Clinical outcomes of ESD for EGCs between useful and useless groups

Clinicopathological features	Useful	Useless	*P* value
Number of cases	42	67	
Complete en-bloc resection	42	59	N.S.
Degree of fibrosis during ESD (F0&F1/F2)	2	12	N.S.
Histologically positive HM	0	5	N.S.
Local recurrence rate (%)	0	0	N.S.
Procedure time (min, mean ± SD)	76 ± 53	95 ± 75	N.S.
Poor control of bleeding During ESD	8	17	N.S.
Perforation	0	4	N.S.
Post-ESD bleeding	1	3	N.S.

*HM* horizontal margin

**Table 3:** Multivariate logistic regression analysis of the variables

Variables	Odds ratio (95 % CI)	*P* value
Atrophic border (Oral side/anal side of tumor)	3.61 (1.18–13.6)	<0.05
Macroscopic type (protruded or flat elevated/depressed)	2.67 (1.13–6.41)	<0.05
Histologic type (intestinal/diffuse)	0.20 (0.01–1.16)	N.S.

## Discussion

In the present study, in 38.5 % cases, chromoendoscopy images with indigo carmine and acetic acid could be used for marking dots around lesions before ESD for EGCs and there were no histologically positive HM cases after ESD among these cases. Our results indicate the number of useful cases diagnosed using chromoendoscopy with indigo carmine and acetic acid. We recognized the limitation of this procedure. As we showed in Fig. 1, the DLs of some cases were clear with the conventional endoscopic images alone, and chromoendoscopy with indigo carmine and acetic acid for these cases were scored “0 (visibility equivalent to that of conventional visibility)”. Therefore, we think that the “useful” cases were only 38.5 % in the present study.

Regarding the useless cases, NBI magnification is necessary for determining the precise demarcation line of EGCs and the clinical course. Kawahara et al. also reported the usefulness of commercialized acetic acid–indigo carmine mixture (AIM) to delineate the margin of EGC accurately [14]. However, the effect of our non-mixture method was not inferior for detecting the DL in cases in which chromoendoscopy was useful. Although the use of both the NBI-ME and chromoendoscopy with indigo carmine and acetic acid are effective for delineating the detailed margin of EGC, some issues needed to be addressed. The usefulness of ME-NBI [13, 23, 24], or ME with acetic acid [25–28], was reported recently for detecting the DL or to observe microsurface/microvascular patterns of EGC. Recently, ME-NBI has been widely accepted in Japan, despite the technical issues that have been identified. First, the scope used in ME-NBI needs to be close to the lesion, and sometimes bleeding occurs from the occasional accidental contact, which subsequently makes it difficult to do a precise endoscopic observation. Secondly, the examination time for ME-NBI becomes longer with the larger lesions. The marking dots before ESD are required in a short time. Thirdly, the ME-NBI needs a dedicated model endoscope. Finally, the endoscopist needs to be skilled in the ME-NBI technique. Conversely, chromoendoscopy with indigo carmine and acetic acid can be performed in relatively short period of time (mean observation time of 71.6 s in the present study), and does not require close proximity to the lesion or special skills. Nevertheless, chromoendoscopy with indigo carmine and acetic acid is not expected to replace the ME-NBI for all EGCs. However, it also has appropriate indications. A simple procedure that confirms the DL in the shortest possible time reduces the endoscopists’ stress. Therefore, we recommend that endoscopists should use this technique primarily for marking dots on the EGCs prior to ESD without ME-NBI. Prospective study is necessary to decide the proper strategy of chromoendoscopy with indigo carmine and acetic acid, and NBI-ME in the near future.

We previously reported on the risk factors for positive HM of EGC resected by en bloc ESD, which included tumor location in the upper third of the stomach and dissatisfaction with the absolute indication for curative ESD [12]. Therefore, local recurrence due to inaccurate the DL determination should be avoided. Tumor location in the upper third of the stomach tends to be difficult to approach with an appropriate distance between tumor and scope without contact bleeding.

The EGC phenotypes were evaluated using CD10 for the intestinal brush border, MUC2 for intestinal goblet cells, human gastric mucin for the gastric foveolar cell, and MUC6 for gastric pyloric gland, respectively. The expression using the mucin phenotype can be more easily and objectively classified using the characteristics of EGCs that are based on intestinal metaplasia. In addition, the mucin phenotype is considered useful for analyzing the correlation between background mucosa and the carcinogenesis of EGCs [29, 30]. There are few reports on the relationship between the mucin phenotype and the DL. Yoshino et al. [31] reported that gastric and intestinal type differentiated adenocarcinomas demonstrate unclear and distinct margins, respectively. There were no reports about the usefulness of chromoendoscopy with indigo carmine and acetic acid according to the different mucin phenotypes. Our results revealed the absence of a significant relationship between chromoendoscopy with indigo carmine and acetic acid and mucin phenotype. In the future, more useful immunohistochemical markers may be evaluated in relation to the tumor carcinogenesis.

In conclusion, to accurately detect the DL, chromoendoscopy should be conducted with indigo carmine and acetic acid, especially for protruded or flat elevated type tumors or atrophic borders on the oral side of tumors, instead of the ME-NBI. A prospective randomized controlled trial involving a larger sample size to compare the ME-NBI and chromoendoscopy with indigo carmine and acetic acid is necessary.

## Conclusions

Before ESD, chromoendoscopy with indigo carmine and acetic acid can be used for creating precise markings in protruded or flat elevated-type EGC or at the atrophic border on the oral side of EGCs.

## Abbreviations

AIM, acetic acid–indigo carmine mixture; DL, demarcation line; EGC, early gastric cancer; ESD, endoscopic submucosal dissection; HGM, human gastric mucin; HM, horizontal margin; JGCA, Japanese Gastric Cancer Association; ME-NBI, magnifying endoscopy with narrow band imaging; PBS, phosphatase-buffered saline
